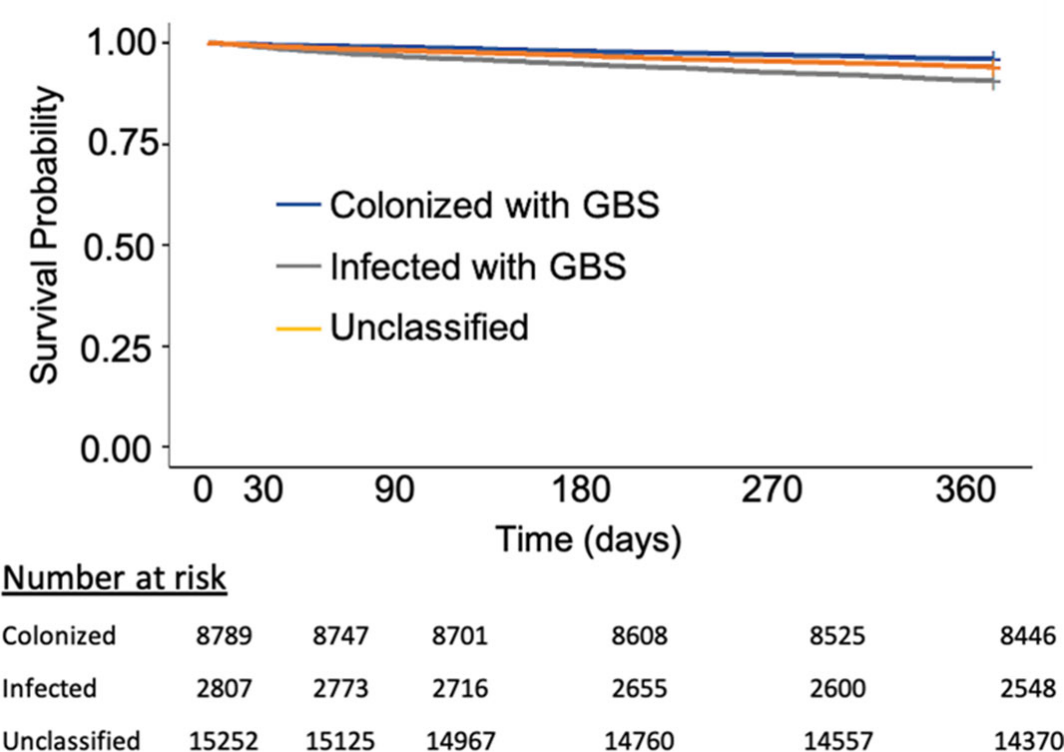# Minimal Mortality Among Veterans with Urine Cultures Positive for Group B *Streptococcus*

**DOI:** 10.1017/ash.2021.67

**Published:** 2021-07-29

**Authors:** Nicole Mongilardi, Robin Jump, Federico Perez, Taissa Bej, Janet M Briggs, Richard Banks, Brigid Wilson, Sunah Song

## Abstract

**Background:** Group B *Streptococcus* (GBS) can cause life-threating invasive infections, yet GBS is also a normal component of the intestinal and genitourinary tract. Although it is regarded as a potential urinary pathogen, the morbidity and mortality associated with recovery of GBS from urine cultures of nonpregnant adults is not well understood. We evaluated characteristics and mortality among nonpregnant adults with urine cultures that grew GBS. **Methods:** Using administrative data from the Veterans’ Healthcare Administration (VHA), we conducted a retrospective cohort study of VA healthcare system users from January 1, 2008, through December 31, 2017, with monomicrobial urine cultures growing ≥100,000 colony-forming units of GBS. Urinary tract infection (UTI) cases were defined as urinalysis positive for leukocyte esterase and pyuria (≥10 white blood cells), an *International Classification of Diseases* (ICD) code for UTI, and an antibiotic prescription. Cases with colonization were defined as negative for leukocyte esterase and pyuria, no ICD code for UTI, and no antibiotic prescription. Cases not meeting either definition were deemed unclassifiable. We compared demographics, comorbidities, and all-cause mortality among these 3 groups. **Results:** Over the 10-year study period, 26,848 veterans had 30,740 urine cultures positive for GBS. Applying the definitions above, there were 2,807 cases of infection, 8,789 cases of colonization, and 15,252 cases that were unclassifiable. Patients with a GBS UTI were slightly older compared to those who were colonized, with a higher Charlson comorbidity index and greater burden of chronic renal disease (Table 1). Individuals with infection versus colonization had 30-day mortality rates of 1% and 0%, respectively, and 1-year mortality rates of 9% and 4%, respectively (Figure 1). **Conclusions:** The association of a greater burden of illness among veterans who met our definition of UTI compared to colonization might be more reflective of providers’ responses to patients with chronic medical conditions rather than a difference in GBS as a cause of UTI. Overall, the prospect of a urine culture that grows GBS does not appear to be associated with adverse long-term outcomes.

**Funding:** No

**Disclosures:** None

**Table 1.**
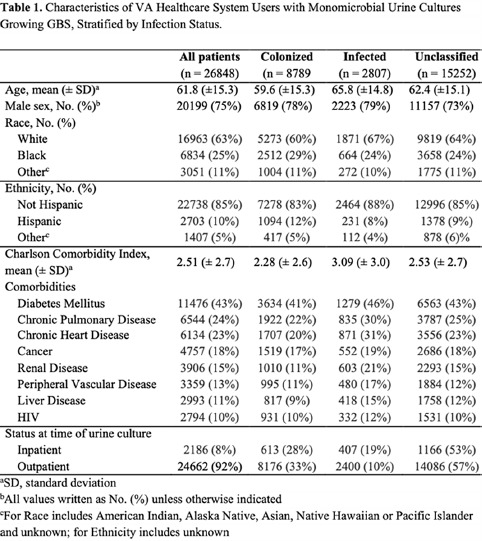

**Figure 1.**